# Lessons Learned From a Physical Activity Intervention in Psychiatric Treatment: Patient, Staff, and Leader Perspectives

**DOI:** 10.3389/fpsyt.2020.00087

**Published:** 2020-03-02

**Authors:** Marit Sørensen, Marte Bentzen, Anders Farholm

**Affiliations:** Department of Coaching and Psychology, The Norwegian School of Sport Sciences, Oslo, Norway

**Keywords:** physical activity, psychiatric treatment, motivation, leader, staff, patient perspectives

## Abstract

**Objective:**

To explore how the implementation of a motivational physical activity (PA) intervention for inpatients with severe mental illness was experienced by patients, staff, and leaders at a psychiatric institution.

**Method:**

After the intervention individual semi-structured interviews were conducted with patients (*n* = 6) and staff (*n* = 6), and a focus group interview was conducted with the leaders (*n* = 4).

**Results:**

All had a positive view on PA as part of psychiatric treatment, thinking it would benefit the patients' health. There were some differences among the groups as to the importance of PA relative to traditional treatments. Positive outcomes were reported from all three groups, but with different foci. The patients and the staff underscored the importance of PA professionals in order to achieve high quality activities, whereas the leaders, due to restraints in resources, could not prioritize to hire PA professionals.

**Conclusion:**

PA was considered a positive part of treatment. Ideas about what it takes to obtain the potential physical, mental, and social benefits of PA differed between patients, the staff involved, and the leaders. Having staff with PA as a primary responsibility and with sufficient competence as PA instructors seems to be important.

## Introduction

Physical *in*activity has been identified as the fourth leading cause of global mortality. It is an independent risk factor for several somatic diseases, such as cardiovascular disease and diabetes (1, 2). On the other hand, physical activity (PA) is widely recognized to prevent a range of disorders, and improve physical health and wellbeing (3–5). In addition, recent systematic reviews and meta-analyses including people with severe mental illness (SMI) have demonstrated benefits of PA on psychiatric symptoms, quality of life, and global functioning as well as on several aspects of neuroplasticity (6–8). The potential of PA is further demonstrated through being found broadly as effective as pharmacological treatment for depression and in preventing mortality due to cardiovascular diseases (9).

There is convincing evidence that people with SMI engage in little PA, especially PA with moderate or high intensity, and they spend a large amount of time being sedentary (10–12). Such behavior can exacerbate the already disproportionally high levels of somatic diseases displayed by individuals with SMI (13–15). Much of the substantially reduced life expectancy in people with SMI can be attributed to these somatic diseases, and in particular cardiovascular diseases (16, 17). Thus, there are compelling arguments for reducing sedentary time and increasing PA in people with SMI.

There are probably multiple reasons for why people with SMI engage in little PA (18, 19). Challenges related to motivation is often stressed as one of the main barriers by both individuals with SMI and mental health staff (20–22). Hence, there has been increasing interest for examining motivation for PA among people with SMI (23, 24). However, few studies have incorporated motivational aspects into their exercise interventions in a structured and theory based manner (25). The trials that have done this, show equivocal results. Some trials yield significant increases in motivational variables but not PA variables, some show significant increases within groups but with no difference between groups, while other show increase in PA variables but not in motivational variables (26–29). Unfortunately, none of these studies described the motivational strategies used or examined how patients and mental health staff experienced the interventions. Thus, there is a need to improve our understanding of how to design PA interventions adapted to the need of individuals with SMI and how these interventions can be implemented into clinical practice.

This study is part of a larger international collaboration on how to increase and implement PA as part of daily clinical practice. The overarching project conducted various PA interventions designed to improve motivation for PA at five different sites (three in Norway and two in Czechia). The study presented here is based on interviews with patients, staff, and leaders from one of these sites. The interviews were conducted to obtain information about the experiences of taking part in a combined 8-week motivational PA intervention. The interviews were performed with these three groups to elucidate the perspectives of all stakeholders in the intervention.

The aim of the study was to explore how the implementation of a motivational PA intervention for inpatients with SMI was experienced from the perspectives of patients, staff, and leaders at one psychiatric institution.

## Materials and Methods

### Design and Intervention

The study had a qualitative design as the aim was to explore the experiences of patients, staff, and leaders at one psychiatric institution. The motivational PA intervention consisted of a planning phase and a two-phased main intervention (baseline and intervention). The planning phase was a collaboration between the research group and staff and leaders at the institution, planning, and preparing the intervention. The two-phased intervention consisted of: 1) an educational phase and 2) implementation of the intervention itself with the patients. The planning and the educational phase took 6 months, and the implementation lasted 6 months.

The educational phase consisted of a 2-h lecture for all staff at the psychiatric institution. The aim was to introduce the project and the importance of PA for the patients. Then an 8-h course was held for staff selected to be co-instructors in the PA-program during the intervention. The aim was to prepare them to gradually take over as instructors, in order to manage on their own after the intervention. Lastly, a 40-h course was given to three external PA-instructors employed in the research project. They all had a sport science education at a master’s degree level. The aim of the course was to prepare them for adapting PA to the needs of this population and deliver the program according to self-determination theory [SDT (30)].

SDT distinguishes between various degrees of internalized motivation for a behavior, ranging from externally controlled (e.g. being told by others) to autonomous (or self-determined, doing it for pleasure). Facilitation of the internalization process is important because the more autonomous motivation is associated with more engagement and persistence of the behavior, and well-being. Facilitation of self-determination is achieved by social environments that support the satisfaction of three basic psychological needs (i.e. need for autonomy, competence, and relatedness) (30). The education of the instructors and staff in the project was focused on how to apply this theory in practice when delivering the physical activities.

Thereafter, the motivational PA intervention was implemented at the institution, led by the external PA-instructors with the selected staff members as co-instructors. For each patient, the participation in the intervention lasted for 8 weeks (1½-week baseline period without PA but with motivational dialogs, and 6½-weeks participating twice a week 60–75 min in the PA program). Once a week the activities took place in a specially equipped activity room at the institution with mostly strength training and a variety of games and play. The typical activity session would start with warm-up activities, then a main strength training part before some fun game or play. The second session of the week consisted of a walk to a rented activity hall a good kilometer away from the institution, a warm-up, and various ballgames. All sessions were closed by a cool-down section with stretching and/or relaxation.” All participating patients were given a wristwatch with an accelerometer in order to record their daily activity.

As the design of the overall research project was a longitudinal multiple single cases design, patients were enrolled in the program continuously over a 4-month period.

### Settings and Participants

The institution in this study offers inpatient treatment for individuals with SMI (e.g., schizophrenia and psychotic disorders, depressive and anxiety disorders, and other). The institution has 30 beds divided between three wards. Normal duration of hospitalization is around 8 weeks. The institution had previously attempted to incorporate PA as an integrated part of treatment without success. None of the staff or leaders had PA or sport science as part of their education. However, two staff members had supplementary training in “PA for mental health care” (30 ECTS).

All participants in the present study were involved in the motivational PA intervention with different roles. The primary role of the leaders (*n* = 4) was to communicate and discuss with the research team and their staff about implementing the intervention and how to organize and support their staff during the intervention. The primary role of the selected group of staff (*n* = 6) was to be responsible for the PA group at the institution and serve as co-instructors during the intervention. The patients' role (*n* = 6) in the project was to be participants in the motivational PA intervention.

In cooperation with the head psychiatrist at the institution the eligibility of the patients for participation was evaluated according to the following criteria: Exclusion: Patients: < 18 years old; performing compulsory exercise as a result of their disorder (e.g., obsessive-compulsive disorders, eating disorders), physical health contraindications, and expected inpatient stay less than 8 weeks. During the intervention period, 22 patients were deemed eligible to participate, whereof 10 patients agreed to participate. One participant dropped out of the intervention due to not finding it enjoyable, while another participant was discharged from treatment earlier than originally planned. Eight participants completed the intervention. Six of them agreed to participate in the qualitative interviews in the present study, which resulted in a response rate of 27% of all eligible patients. All staff members involved in the project/intervention (*n* = 6), and the leaders (*n* = 4) agreed to participate in this study.

### Data Collection

#### Background Data Collection

In cooperation with the staff at the institution, patients' gender, age, and diagnoses according to ICD-10 were registered. Staff and leaders were asked to register their age, gender, occupation, and years of experience working in psychiatry in general, and at the current institution specifically.

#### Qualitative Data Collection

After the intervention period had finished, individual semi-structured interviews were conducted with patients and staff, while a focus group interview was conducted with the leaders of the institution. All interviews took place at the institution in a quiet and undisturbed room to facilitate a safe and comfortable environment for the participants to share their experiences (31). The individual interviews were conducted by the second author, while the focus group interview was conducted by the first and second authors. All interviews were audiotaped. Both interviewers had a central role in the research project, one as project leader and one as an employed researcher. They were thus well familiar with all phases of the project, and had been engaged in several meetings with both leaders and staff prior to the interviews. As for the interviews with the participants, the interviewer had not met the patients before the interviews.

A summary of the interview guides (available on request) reflects topics of importance in order to shed light on the experiences of the participants, the staff, and the leaders with the motivational PA intervention. Central topics for all three populations were questions regarding their relationship and experiences with PA as part of treatment, experiences with the instructors/being instructors, motivation for PA, and motives for participating.

### Data Analysis

The data analysis followed Braun and Clarke's six-phase approach for thematic analysis (32): 1) Familiarizing with the data, 2) generating initial codes, 3) searching for themes, 4) reviewing themes, 5) defining and naming themes, and 6) producing the report. Phase 1–4 will be described in the method section, while phase five is described in the result section, and phase six is the discussion. All three authors contributed to phase one, four, five, and six. Phase two and three were conducted by the second and third authors, and thereafter discussed by all authors.

The interviews were transcribed verbatim by a research assistant, resulting in 159 pages of single-space raw text (53 pages for the patients, 85 pages for the staff, and 21 pages for the leaders). The qualitative analysis software MAXQDA was used when manually coding the data. The authors got familiar with and immersed in the whole set of data when they thoroughly read it at least twice. During this process, initial ideas and comments were noted by each of the authors while reading. At the end of this phase, all authors met and shared their initial impressions of the data.

One researcher progressed to phase two and generated initial codes of all the data based on interpretations of data of relevance for the research question. During this phase, data were coded both semantically and conceptually, by reading between the lines (33). During phase two, the second author who had been involved in all the interviews served as a discussion partner being able to share additional information about impressions of attitudes and emotions underlying the transcribed text. Further, the analytic process lead to phase three as these two authors started to search for themes when looking for meaningful patterns of data relevant for the research question. To guide this process, both maps and tables of codes and themes were drawn on paper by the researchers. Themes were refined, combined, separated, and discarded during this process (32). At the end of this phase, suggestions of themes for all three levels of the data (patients, staff, and leaders) were set.

At the fourth phase, the first author re-joined the process serving as a critical friend (30) which fuelled the progress of reviewing the themes. Central elements of this phase involved reviewing whether and how the coded data extracts and themes reflected the full data set (32). This process of discussing and reflections about possible interpretations of the data were done to counteract possible biases within the process of qualitative analyses (31). This process lasted until the three authors reached consensus about the refinements for each theme and the overall story of the whole dataset, which contributed to increase the trustworthiness and credibility of the findings (34).

### Ethical Considerations

A code of ethics was outlined for the project based on the “Meta-Code of Ethics” from The Federation of Professional Psychologists´ Associations (35) including these main principles: respect for the rights and the dignity of the participants' competence, responsibility and integrity. These principles were followed during the outline of the project, in cooperating with all involved staff at the institution, during the intervention in all activities with participants, and in the process of collecting, storing, and analyzing data. A written agreement was developed and signed by the psychiatric institution and the research institution. These ethical matters also involved confidentiality of patients, staff, and leaders, and they all signed a written informed consent to participate in the study. The study was approved by the Norwegian Regional Ethics Committee South-East (2015/1536).

It is important to emphasize that the PA-instructors that were educated to implement the PA-intervention only served as “additional personnel” in the physical activities. These PA-instructors did not have activities on their own with the patients, staff from the institution were always present. The instructors signed an agreement of confidentiality with the institution.

In order to ensure anonymity, all information that could identify the participants were kept locked in a safe in the office of the leader of the institution. Unidentifiable data were kept in a designated computer without access to the internet, stored in a locked cupboard in the office of the researchers.

## Results

### Participant Characteristic

In total, 12 individual semi-structured interviews were conducted (six patients and six staff members) and one semi-structured focus group interview (four leaders). **Tables 1** and **2** show the characteristics of the participants.

**Table 1:** Participant characteristics, leaders and staff.

	Leaders (*n* = 4)	Staff (*n* = 6)
**Gender**	Female (4)	Female (3) Male (3)
**Age range**	43–58 years	28–43 years
**Profession**	Psychologist (1) Specialized nurses (3)	Milieu therapists (3) Specialized nurse (1) Nurses (2)
**Work experience in MHC**	10–14 years (2) 15–19 years (1) 20–24 years (1)	1–5 years (2) 6–15 years (3) >20 years (1)
**Experience as leader in MHC**	5–9 years (1) 10–14 years (3)	

MHC, mental health care.

**Table 2:** Participant characteristics, patients.

	Patients (*n* = 6)
Gender	Female (4) Male (2)
Age range	21–44 years
Diagnosis group (primary)	F20–F29 (1) F30–F39 (2) F40–F48 (2) Not reported (1)
Attendance rate MPAI (max 14)	1–5 (1) 6–10 (3) 11–14 (2)

### Findings

**Figure 1:**
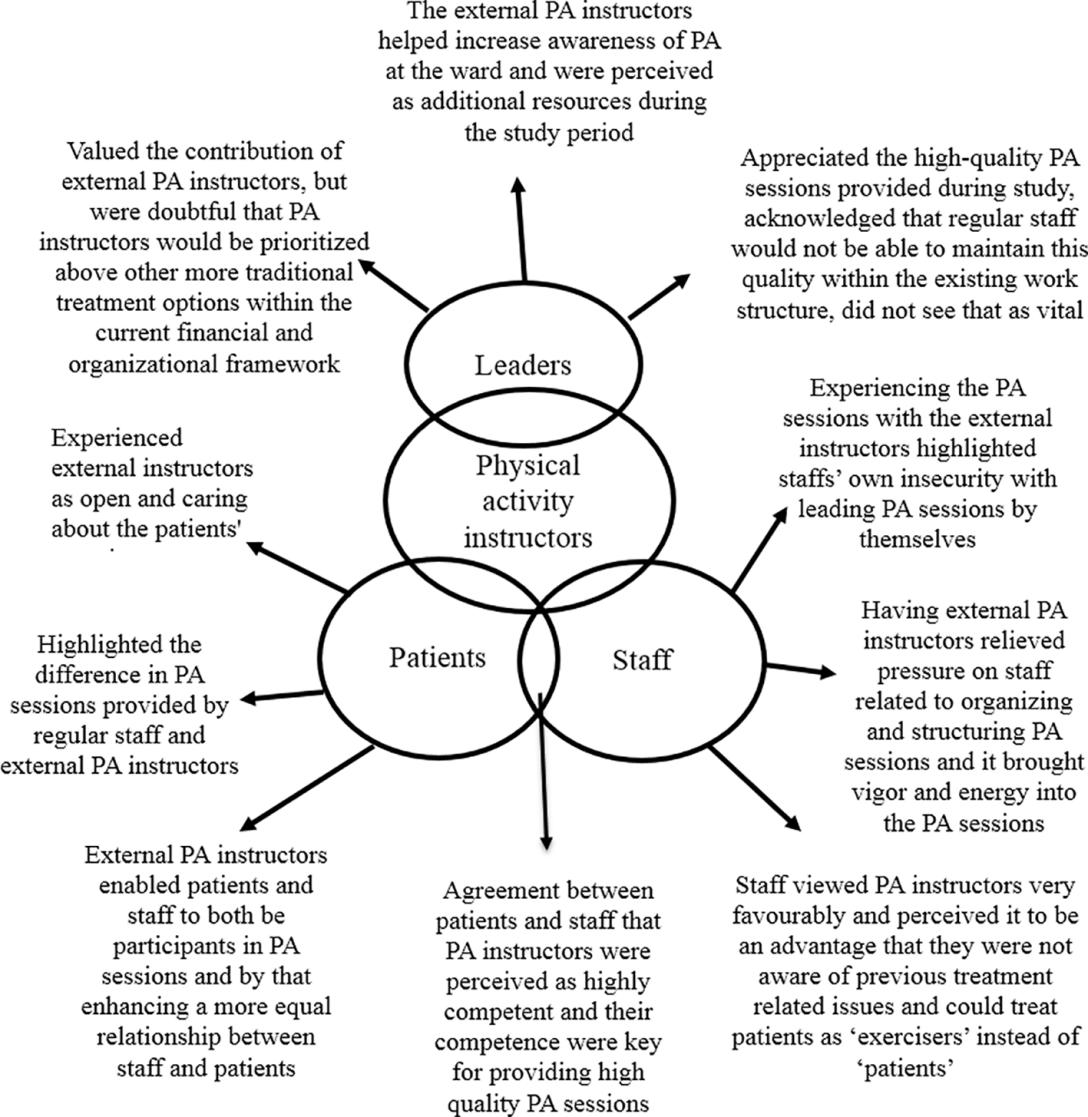
Example of variations in opinions between the groups on the topic of physical activity (PA) instructors.

The analyses resulted in 1,433 codes that made out seven main themes. The findings presented below are structured around the five main themes related to the aims of this article, the remaining two themes were more general descriptions of how the institution was organized and worked, and the background of the staff. In order to fully take advantage of incorporating patient, staff, and leader perspectives of the intervention, findings from all interviews are presented together. Quotations from leaders are marked with L, staff are indicated by S, and quotes from patients are marked with a P, and a number indicating which person. Within each theme, concordance and discrepancy between the three perspectives are highlighted and they will be discussed later in the paper. An example of a theme is displayed in **Figure 1**, and an overview of the main themes and core findings for patients, staff, and leaders is displayed in **Table 3**. Naturally, there were variations in how much the three perspectives contributed to each of the themes.

**Table 3:** Overview over themes and core results from the three populations.

Main themes	Core findings for each population		
	Patients	Staff	Leaders
Viewpoints on PA as part of treatment	Positive attitude toward PA	Positive attitude toward PA—BUT variation related to own PA experience	Positive attitude toward PA—BUT pragmatic attitudes due to limited resources and traditions
Motivation for involvement and the PA	Motivation to get in better shape, start expectations of positive physical and mental effects	Mixed—some highly motivated, some felt pressured	Wanted more PA for the patients, saw project as additional resources and help
Experiences with PA as a part of treatment	Positive experiences both physically (although strenous at times), mentally and socially	Conflicting experiences due to competing responsibilities at the ward and lack of own experience with participating and leading PA	Important supplement BUT—financially and organizationally challenging after the project
Outcomes and experience of the PA and the intervention	Mostly positive Varying increase in PA and motivation Positive patient–staff relationship	Mixed experiences Some wanted to continue, others withdrew Positive patient–staff relationship	Increased awareness of PA, helped structure and support
Experiences with external PA-instructors/own instructors	Very good experiences with external instructors, appreciated competence and them being “neutral”	Very good experiences with external instructors, very mixed as to own competence	Positive to external instructors but cannot prioritize the resources in the further

#### Viewpoints on PA as Part of Psychiatric Treatment

All three groups had a basic positive view on using PA as part of psychiatric treatment. For the patients, PA organized at the ward was a valuable opportunity to improve their fitness and it was described as a “kick-start for becoming more physically active again”. They had all taken part in various physical activities in different phases earlier in life.

Well. I know myself that I feel better when I am physically active. Firstly, if I am not active, I struggle with bad conscience towards myself, that I have not managed to do it. So simply to be active helps a lot in that. But with physical activity I also get better sleep. I eat more and better because I need it in a totally different way, eehm… so the physical activity helps me a lot with other things that are difficult. But then it is also difficult when you are really shitty… then it is difficult to manage the physical activity, and it gets a little heavy (P3).

The staff were in agreement that PA could be beneficial for patients as part of a holistic treatment approach. Furthermore, the staff emphasized that PA could have positive impact on both physical and mental health and that it was “*an activity where one could feel a little normal for a while”* (S5). However, the staff also acknowledged that in a short treatment stay, PA competed with many other aspects of treatment often considered to be more their primary responsibility (e.g. medication, social therapy, economy, psychotherapy, etc.).

Yes, for example you schedule to participate in the activity lesson, prepare for it, motivate the patients for days, and in comes a message “from above” that a new patient is arriving at that very time. And then the two crash, and you have to choose, and that is unfortunate, but no days are completely planned. That was just one example, but that is how the days are. There is a lot in and out and there are appointments that do not…, and then there is no concern for the activity project, if you see (S3).

For patients with complex problems they also often had to prioritize some challenges over others. In sum, the staff viewed PA as a valuable contribution to the treatment, but with the current treatment guidelines it would often be given lower priority in conflict or competition with other forms of treatment.

Similar to the staff, the leaders also perceived PA useful as a part of a holistic treatment approach. PA was also described as both a means and a goal, meaning that PA, in addition to the physical benefits, could be used as a means to regulate feelings or other behavior. Leaders also felt that being part of the present study helped increase the focus on PA at the institution, but at the same time they acknowledged that PA was an aspect of the treatment that often would be neglected because it was not yet as incorporated in the daily routines as other more traditional treatment strategies. Challenged about whether they considered PA to be a form of treatment on a par with the traditional treatments in use, some uncertainty surfaced:

If we try to be completely honest whether we have considered physical activity as treatment, or whether it is a supplementary thing that is nice to do, but that is not necessary to do, ehh, there may be an element of that, too (L4).

#### Motivation to Take Part in the Project and the PA

The patients described motivation in two ways, namely, motivation to take part in the project and to *attend* PA, and motivation *during* PA, which is more an outcome. Motivation to take part and attend PA was fuelled by expectations of PA leading to improved mood, fitness, and sense of well-being, and that it was good for them.

Yes, I want to! In that meeting they mentioned that maybe this project could be something, or is it a little premature?” But then I thought.no, it is not, I need to get started. Of course I know that, I want it, really, even if I feel (pressured?) by the environment… (P6).

I have never tried or taken part in anything similar before. So it was a little…I did not know what I went for, but thought that it surely would be good for me (P1).

However, the patients described motivation as fluctuating and that it could be challenging to be motivated to attend PA. Interestingly, three of the patients had a quite clear perception of motivating themselves, rather than the staff motivating them, when they felt it was difficult to attend PA sessions.

During the study period, the staff viewed the “motivational work”, were they encouraged patients to participate in PA, to be a major and demanding work task. Several of the staff described that doing this on their own without support from other colleagues would not be sustainable in a long-term perspective. The staff also agreed that personal interest and considering PA as a valuable part of treatment to be pre-requisites for doing efficient motivational work. However, the staff did not see it as necessary that they themselves were responsible for the PA sessions. In fact, having highly competent external instructors was perceived to facilitate the motivational work at the ward. Finally, the staff found it easier to motivate patients to attend PA later in the project because it took some time before they were able to incorporate PA as a natural part of their work assignments.

For their own participation, the motivation varied with personal interest in and experience with PA. Some felt pressured by duty and felt uncertain and uncomfortable in the role.

I was simply asked. Eh, I got the impression that there was nobody else to ask, and then you get a little forced in a way, because… but I do not have anything against it. I am interested in PA and such things, but somewhat forced, I felt it was inevitable, and… (S4).

The leaders wanted more PA for their patients, and was motivated to take part in the project by the possibility to get additional resources to increase awareness about PA and help establishing PA at their institution.

Eh, we have focused on trying to get PA started through many years, and know that it is difficult with rotation schedules, so I wanted very much that we could try to succeed. So I was positively interested (L2).

They also focused on that the patients should continue with PA after being discharged from the ward. Thus, they acknowledged the importance of motivation, but they also highlighted that most patients would be in need of some sort of support to maintain their PA routines when transferred to community mental health care. With regards to their staff, the leaders perceived them to be more assured in their motivational work because they had confidence in the PA sessions being of high quality during the project. Further, they expected the staff to learn enough to take over the responsibility after the project period.

#### Outcomes and Experience With the PA and the Motivational PA Intervention During the Study

##### The PA

In general, the patients had positive experiences with PA during the study period. They highlighted that PA was enjoyable and that it was a welcomed opportunity to do something else than the everyday routines at the ward. They reported that it was easier to be physically active when less ill, but also if they managed to take part when ill, it made them feel better, both during the activity and some time after. The patients underscored the importance of a fixed PA schedule rather than having to take initiative themselves, because it made it easier to take part in PA.

Yes, it was sort of a small break. And then it was a fixed schedule, so that you knew when it was and not so that you had to make up your mind to do this and that, it was in a way somebody else who had it organized, so all you had to do was to show up. That made it a little easier (P1).

The patients related that the motivating factors *during* PA were feeling better, learning and mastering various PA exercises, getting informational feedback, PA being fun and enjoyable, getting started to be physically active again, and having highly qualified instructors leading the PA sessions. Effects in close proximity were said to be most motivating [i.e. feeling better (e.g. no anxiety) during and right after PA, and performing better than last session]. Moreover, it was both the physical aspects of PA (i.e. activating the body and improving fitness), mental effects (e.g. no anxiety during activity and a “feel better/more relaxed” effect), and social (i.e. meeting and talking to other patients outside the ward and building relations with the staff) that made it a positive part of the treatment.

If you are physically active you are together with people without having to talk so much, in a way. And you get a sense of community all the same (P2).

Of notice is that the patients had differing preferences of the PA content (i.e. some patients favored team and ball sports while others tended to prefer individual activities such as circuit training), and that the content and organization of PA sessions influenced the experience patients had of PA. Lastly, the patients mentioned that the physical activities sometimes were experienced as strenuous and that they had to take breaks or adjust their effort. However, the patients typically did not describe being tired from PA as particularly negative, they seemed to like the feeling of having had a good workout. It was also an opportunity “to do something normal”.

The staff agreed among them that the patients had positive experiences from taking part in the PA sessions. For their own part, the experiences were more mixed. Some of the staff viewed it as mostly fun and enjoyable, and one even mentioned that taking part in PA removed some of the psychological exhaustion he felt when working so closely with people inside the ward. On the other side, some also reported that taking part in PA was physically exhausting and that it was challenging to be tired and uncomfortable in front of the patients. For one of the staff, taking part in PA was so exhausting that it sometimes felt problematic to get back to work after the PA session.

..and you see, I am not very physically fit, so when I got back to work I was so exhausted that it was not realistic for me to continue working…. However, this is about my poor fitness, but that combination…, yes all I wanted was to go home and to bed! (S1).

The staff experienced the motivational PA intervention as a way of legitimizing using time and resources on promoting PA as part of treatment. They also described that the intervention had raised the general awareness of PA at the ward. On the other hand, several of the staff perceived taking part in the intervention, and in particular taking on the role as a PA instructor, so demanding that they withdrew from having an active role in PA after the conclusion of the project.

The leaders did not have a first-hand experience of the PA sessions. They mentioned what experiences they thought patients and staff had from taking part in PA, based on conversations and reports. As to patients, the leaders thought the patients had positive experiences of taking part in the PA sessions. As to the involved staff, the leaders acknowledged that the personal relation and previous experiences of PA and exercise among the staff probably influenced to which degree staff had positive experiences of taking part in PA sessions. Moreover, the leaders also linked this study to another project at the ward were staff were encouraged to walk more and the leaders thought some of the staff used the current study to change their own PA behavior. The major outcome from the leader perspective was how the motivational PA intervention helped increase awareness of PA among staff and that it gave them structure and competent support toward integrating PA as part of the overall treatment at the hospital. That being said, the leaders planned to downscale the PA offer after study conclusion in order to secure a sustainable extension of PA at the wards.

##### Staff-Patient Relationship During PA

Taking part in the PA together generated predominantly positive experiences. The patients enjoyed participating together with the staff, and it was seen as positive for the patient-staff relationship. It also felt safe when meeting the external instructors in the beginning:

It was very good, because in the beginning when I did not know the instructors, I knew those who worked here, and it felt a little more safe, in a way (P4).

However, it had happened that the staff could forget their professional role during the activities.

But sometimes….one forgets that a patient may be at a different level when it comes to activity, so it is important, in particular during ball games, to remember that…….It may be difficult, but to keep people in the game when you participate and not be overrun by those who work here, for example (P5).

Similar to the patients, there was consensus among the staff that taking part in PA together with patients could facilitate a positive staff-patient relationship.

….you got to know each other in a different way through physical activity, and we had more to talk about and an easier way to get a conversation going in everyday life, and because they had that watch (with the accelerometer) that was absolutely positive (S3).

However this could happen at the expense of being comfortable in a professional role:

Ehh, I very clearly got the feeling that I was the one who could not handle it, to put it that way. At that time we were not so many, it was only me and one (patient) from our ward. So we had it as a common thing among us, that we both had our challenges, so in that way it was a good thing, Eeh, personally I thought it was really awful (S1).

This topic did not emerge in the leader interview.

#### Physical Activity Instructors

The external instructors were highly regarded by all interviewed participants and it was unanimous agreement between patients, staff, and leaders that they were a key to organizing high quality PA sessions.

From the patient perspective, the competence of the external instructors was displayed by how they were able to adjust PA sessions according to the patients’ preferences and unexpected events (i.e. many or few participants, change in location). The patients also appreciated that they could give informational feed-back on technique, adjust specific exercises to fit their skill/fitness level, provide variation in content, helping them learn new skills, and their ability to organize fun and engaging PA. The external instructors were also viewed as nice, friendly, and respectful in their interactions with the patients.

I think, I got a very favorable first impression at least. I think they seemed very down to earth and took our training seriously. Good structure and so on in all we did, and what instruction that was given and so forth. And then I think, yeah… it was easy to get to know them. It was… they seemed quite open, yea… (P1).

The staff shared the patient perspective of the external instructors as highly competent and emphatic. When directly asked about whether there were any disadvantages with external instructors, none of the staff mentioned any drawbacks. On the contrary, staff highlighted that because the instructors were external, their interaction with the patients were not colored by diagnoses or issues related to other aspects of the treatment. It was easier for them to treat the patients more like “exercisers” than “patients”. Moreover, the staff agreed that the external instructors brought energy and knowledge into the PA sessions. The promotion of PA at the wards was made easier by keeping up regularity and structure to the PA.

The leaders had a more organizational view on the external instructors. They were in favor of using external instructors because it added resources to the institution and it helped increase the awareness of PA at the wards. They also recognized that lack of staff having PA as a first-priority work task was one of the reasons for previous failures to implement PA as part of treatment. However, from a financial and organizational view, they doubted that external PA instructors would be prioritized after the study period. Organizing and leading PA was viewed as a task that the regular staff should be able to perform. Even though they could reorganize and find funding for a small PA instructor position, they argued that such funding should rather be used to hire a psychologist, yet again demonstrating a view of what is the more important form for treatment.

Although the staff functioned less as PA instructors during the study period than planned, the patients were quite clear in their opinion that the regular staff did *not* have the competence needed to deliver high quality PA. One of the patients exemplified this by stating: *“the staff working here do not have that kind of education, but I am sure they can learn”* (P2). Another said:

I think there should be more focus on the staff generally in order to change attitudes so that physical activity is a part of the therapy and not only something isolated. So there must be a change in attitude if they are going to be able to run it themselves, without you (external instructors) (P1).

On the other hand, the patients were in favor of doing PA together with the staff, mostly because it created a feeling of relatedness and equity, but also because it gave a sense of security when meeting the unknown external PA instructors in an unfamiliar situation.

The study protocol specified that staff assigned to the co-instructor role were supposed to take over the responsibility for leading the PA sessions halfway through the study period. However, the staff did not feel ready or competent enough to lead the PA sessions, thus, the external instructors had the main responsibility for PA sessions throughout the study period. This feeling of insecurity was reflected in the answers of the staff when asked about their feelings of being responsible for PA at the ward. This insecurity and sense of incompetence resulted in some of the staff ending their involvement as PA instructors after the intervention finished. Others were willing to continue being involved in PA. This was due to a combination of perceiving PA as an obliged work duty and not considering other colleagues any more competent to be a PA instructor. However, if they were to be responsible for the PA sessions, it had to be adjusted according to their own level of “PA instructor competence”.

The leaders had a two-dimensional perspective on staff as PA instructors. They agreed on the need for a certain level of competence to deliver high-quality PA sessions. However, the leaders thought that taking part in the study should have enhanced the ability of the staff as PA instructors. At the same time, they acknowledged that the staff were still not able to deliver PA with the same level of quality as in the project. However, the leaders doubted that it was possible, both organizationally and economically, to extend the organization of PA as it had been during the study. With this as background, the leaders suggested that it would be more feasible to offer “movement” that did not require as much competence from the staff, “like walking or a 10 minute morning bend and stretch”. By lowering the expectancy of what the staff would have to do when leading and delivering PA it would, according to the leaders, be more realistic to incorporate PA as part of the overall treatment at the wards.

## Discussion

PA was experienced as beneficial by all three groups that were interviewed. For the patients, it should be taken into consideration that because participation must be voluntary, the participants only made out around one third of those who were eligible. Hence, it is likely that they were a select group of motivated individuals. This group clearly wanted PA as part of their treatment, and some even commented that the attitudes toward PA among some of the staff in the institution prevented this. Again it must be remembered that this most likely is not representative for all the patients that did not want to participate in the project, even if some of them also took part in the activities now and then. However, the patients demonstrated being well aware of the health benefits of PA, both theoretically and from own experience.

The participating staff were also positive toward including PA in the treatment regime, and perceived that it benefitted the participants. However, they experienced frequent conflicts with other tasks perceived to be more their primary responsibility. Therefore they felt they needed support from either external instructors or to have someone among them with PA as the primary responsibility.

The leader group wanted more PA for the patients. The very reason for joining the project, was a hope that it would help getting PA more firmly established in the institution. However, with tight budgets, a hectic work situation and complicated rotation schedules, PA would lose a competition against the more traditional therapies.

All in all, this situation is probably not unique to this particular institution. Individuals with psychiatric illness often want to become more physically active (36). However, perceived barriers and the quality of their motivation make them need help, support and high-quality activity possibilities (37, 38). The situation for the staff is supported by a few other studies. Glowacki, Weatherson, & Faulkner found in a scoping review of mental health care providers that beliefs that the patients could not overcome barriers to PA engagements, and a lack of training on how to promote PA to individuals with mental illness were the most common barriers to PA promotion (39). Also, a study including 219 health personnel in seven psychiatric institutions demonstrated that the traditional health professionals had positive attitudes toward PA for psychiatric patients, but did not feel that it was their area of expertise or their responsibility (40). The knowledge about the important role of PA for both the physical and mental health that has been generated only during the last decade is obviously still not commonly known in our health care. Such dissemination takes more time (41).

Some of the patients felt that the staff had helped them to keep up motivation to take part in the PA, others felt that they mainly had motivated themselves. This may partly be due to the fact that even if the study protocol was that the motivational dialogs during baseline should be conducted with the external instructors and one of the staff together, very few of the staff actually took part in these dialogs. Because the basis for the motivational strategies were supposed to be established during these dialogs, missing out on these meetings may have made the motivational work more challenging for the staff at times, and less efficient.

The motivation among the staff varied, some wanted to continue and take over the responsibility as instructors, whereas others took part solely out of a sense of duty or even felt pressured. This is of course partly due to their personal relation to PA, but with better and more thorough information about the task, in particular the fact that they were intended to take over as instructors during the study period, some frustration could have been avoided. However, it is not to be expected that all health care personnel see it as their job to work with PA, because this is not so far a prominent theme in their education, and has probably not been part of the job description of the position they were employed in.

Both patients and the staff were generally satisfied with the activities, and described positive physical, mental, and social outcomes, with a very few exceptions. For the patients this is obviously colored by the voluntary nature of taking part in the study. Choosing to participate probably means to be motivated to do PA. The fact that some of the staff felt pressured to participate and felt uncomfortable in doing it, would naturally affect their experiences. Blanner Kristiansen et al. (38) found that if staff with negative attitudes toward physical activities engaged in PA with the patients, this could serve as a barrier for integration of PA in psychiatric treatment. This may be an important issue for the leaders to take into consideration when they expect the regular staff to be able to run sustainable PA in their institution. Both patients and staff described outcomes of the PA in more dimensions and in more detail than the leader group, which most likely is a result of the concrete participation in the activities.

Taking part in a research project added awareness, weight, and significance to the PA.

It added commitment and enthusiasm for those taking part, even if it was a novelty for them to record what they were doing by accelerometers and questionnaires. However, it must be acknowledged that two thirds of the eligible patients did not want to take part in the study. Although, some of them participated in the PA sessions at times.

The external instructors were perceived positively by all involved parties due to solid competence and an open, empathic style of leading the activities. It should be noted that these instructors were master students of sport science with additional specialized education. They had all some work experience from psychiatry as well as a 40 hours special preparatory course within this project. The patients and the staff appreciated their skills and knowledge, the patients felt they were taken seriously, and the staff valued the support and the responsibility they lifted off their shoulders. The patients valued in particular their ability to individualize, give feedback, structure, adapt and find fun activities, and did not find those qualities well enough developed among the regular staff. The staff themselves largely agreed, some feeling that they did not have the necessary competence. This is supported by a study demonstrating that PA improved cognitive functions significantly when supervised by PA professionals, whereas those supervised by other professionals (i.e. mental health support) did not (42).

Again, the leaders had a more pragmatic approach colored by budget and resource restraints. They thought that the health personnel ought to be able to run the activities themselves, and if necessary settle for simpler activities such as walking. This underestimation of what high quality PA is and means for the patients, and what competence it takes to deliver it, is also likely due to not experiencing this difference in the activity setting for themselves as well as the limited resources.

### Strengths and Limitations

The strengths of this study is that we were able to register the experiences of all parties that had been involved in the intervention, and obtain an understanding of the different perspectives. A limitation is that the patients were a select group, as only one third of the eligible patients volunteered to join the project. Due to the voluntary nature of participation, those motivated and with former PA experiences took part in the project, even if other patients sometimes took part in the PA.

This study was conducted at a relatively small institution, with limited facilities for PA. However, as a part of the project we tried to make most out of the facilities, like clearing one room for an activity room, investing in sport equipment, using the garden, and renting a sports hall once a week. Most psychiatric institutions may not have good PA-facilities, and as this intervention aimed to be an effectiveness study (43), it was of importance to explore whether such an intervention could be effective in an “ordinary” institution.

The knowledge and engagement of the interviewers in the implementation of the intervention can be considered to be a methodological strength as the good relation to the staff and leaders made it easier to go into depth about experiences about the intervention. They were aware of situations that had been perceived as successes, but also the challenges. On the other hand, this could also make the researchers biased.

Eight weeks is a relatively short period of time for an intervention aiming at motivational and behavioral change in PA. The short duration of stay for the patients also resulted in a somewhat hectic treatment regime, raising important questions regarding priority between different aspects of treatment for each patient. This might have influenced the experiences reported by all three groups of participants in the study.

Difference in data collection methods between the groups, with individual interviews of patients and staff, and a focus group interview with the four leaders, may also have influenced discussions and the themes brought up during the interviews differently.

### Implications

Leaders, staff, and patients expressed positive views on including PA in a psychiatric treatment stay. However, there were different ideas between patients, some of the staff, and the leaders about what it takes for the patients to obtain the potential physical, mental, and social benefits of the PA. Based on former attempts to offer PA at the institution, and this project with external instructors, it seems as if having staff with PA as a primary responsibility and with sufficient competence as PA instructors is a key to succeed.

## Ackowledgments

We would like to thank the participants and staff in the psychiatric center for their efforts and enthusiasm through this project. We would also like to thank our colleagues in The Czech Republic for their efforts in applying for the grant and collaboration in making the total project a reality.

## Data Availability Statement

The datasets generated for this study are available on request to the corresponding author.

## Ethics Statement

The Regional Ethical Committee for Medical and Health Related Research (South East Norway) approved this study. Written informed consent was obtained from all participants for their participation and the publication of their personal data.

## Author Contributions

MS was the PI of the project and was active through all phases. MB took part in design and planning of the project, had the main responsibility for collecting data, and took active part in writing of the manuscript. AF had the main responsibility for the data analyses, and took active part in writing of the manuscript.

## Funding 

The study was performed with the kind support of the Norwegian Grants, Czech-Norwegian Research Program (CZ09), project ID Number: 7F14500 during the years 2014-2017.

## Conflict of Interest

The authors declare that the research was conducted in the absence of any commercial or financial relationships that could be construed as a potential conflict of interest.
